# Knowledge, attitude, and practice toward weight management among diabetic patients in Qidong City, Jiangsu Province

**DOI:** 10.1186/s12889-024-18392-2

**Published:** 2024-03-29

**Authors:** Xiaofeng Li, Yu Shi, Dongqin Wei, Yan Gong, Xinyi Yan, Shengnan Cai

**Affiliations:** Department of Endocrinology, Metabolic Managament Center, Qidong People’s Hospital, Qidong Liver Cancer Institute, Affiliated Qidong Hospital of Nantong University, 226200 Qidong, China

**Keywords:** Knowledge, Attitude, Practice, Diabetes mellitus, Weight management, Cross-sectional study

## Abstract

**Background:**

Weight management is an effective prevention and treatment strategy for diabetes mellitus. This study aimed to assess the knowledge, attitude, and practice (KAP) of diabetic patients towards weight management.

**Methods:**

Diabetic patients treated at Qidong City, Jiangsu Province, between January 2023 and June 2023 were included in this cross-sectional study. A self-designed questionnaire was used to collect their demographic characteristics and KAP toward weight management. Structural equation modeling (SEM) was employed to examine the inter-relationships among KAP scores.

**Results:**

Among a total of 503 valid questionnaires that were collected, 55.07% were filled out by men and 54.67% by those aged < 60 years. The mean scores for knowledge, attitude, and practice were 8.03 ± 3.525 (possible range: 0–13), 31.88 ± 3.524 (possible range: 10–50), and 22.24 ± 3.318 (possible range: 9–45), respectively. Pearson’s correlation analysis revealed the knowledge was positively associated with attitude (*r* = 0.295, *P* < 0.001) and practice (*r* = 0.131, *P* < 0.001), and attitude was positively associated with practice (*r* = 0.140, *P* = 0.002). SEM demonstrated positive associations between knowledge and attitude (β = 0.28, *P* < 0.001), and attitude and practice (β = 0.09, *P* = 0.019). Moreover, older age was negatively associated with knowledge (β=-0.04, *P* = 0.001), while higher education (β = 1.220, *P* < 0.001), increased monthly income (β = 0.779, *P* < 0.001), diagnosis of fatty liver (β = 1.03, *P* = 0.002), and screening for excess visceral fat (β = 1.11, *P* = 0.002) were positively associated with knowledge.

**Conclusion:**

Diabetic patients showed moderate knowledge, neutral attitudes, and inappropriate practices toward weight management. Knowledge was positively associated with attitude and practice. These findings provided valuable directions for healthcare interventions targeting improved KAP status of weight management among diabetic patients.

**Supplementary Information:**

The online version contains supplementary material available at 10.1186/s12889-024-18392-2.

## Background

Diabetes mellitus is a chronic metabolic disorder characterized by hyperglycemia caused by insulin secretion or action defects, which came to pose a substantial global health concern [1]. The escalating prevalence of diabetes has prompted its classification as a modern pandemic throughout the world [2]. According to The International Diabetes Federation (IDF), there were some 463 million adults living with diabetes in 2019, and this number is expected to reach 700 million by 2045 [3]. Specifically, China has the largest diabetic population globally, with approximately 116 million individuals affected as of 2019 [3]. Diabetes mellitus has a multifaceted impact on individuals, transcending mere physiological derangements. First, diabetes exposes patients to debilitating complications, including cardiovascular diseases, neuropathy, retinopathy, and nephropathy [4, 5]. Second, the psychological toll is equally significant as patients manage self-care demands and cope with chronic illness distress [6]. Therefore, the confluence of physical and psychological effects emphasizes the urgency of robust preventive measures and comprehensive management strategies.

Given the far-reaching consequences of diabetes mellitus, its prevention and management are of paramount significance. Among the promising strategies for managing diabetes mellitus, weight management is a practical approach, encompassing diverse interventions like dietary adjustments, physical activity, and behavioral changes directed toward achieving and sustaining a healthy body weight. Effective weight management practices among diabetic patients yield notable advantages, such as heightened insulin sensitivity, enhanced cardiovascular health, and improved overall well-being [7, 8]. Crucially, weight management has a central role in diabetes prevention, facilitating clinical remission and diminishing complication risks [7, 9]. Behavior therapy has been utilized to foster healthy dietary practices and weight reduction among overweight or obese individuals [10]. This psychotherapy highlights systematic intervention to modify maladaptive behaviors. However, awareness of conducting weight management, as well as its preventive roles in diabetes, are crucial in mitigating the risk of diabetes. Moreover, a positive attitude can bolster an individual’s confidence in weight management and contribute to elevated compliance. Therefore, understanding the KAP status regarding weight management among diabetic patients can have implications for efficacious strategies. For instance, prior research from Iran and South Africa revealed limited awareness of weight management benefits in diabetic patients [11, 12]. Despite an expanding literature on diabetes mellitus, there is a considerable dearth of KAP studies in China, especially those focusing on weight management among diabetic patients. Therefore, this study aimed to assess the KAP towards weight management among diabetic patients in Qidong City, Jiangsu Province, China.

## Methods

### Study design and patients

This cross-sectional study used convenience sampling to recruit diabetic patients in Qidong City, Jiangsu Province, between January 2023 and June 2023. Inclusion criteria were: 1) diagnosis of type 2 diabetes (T2D) according to the World Health Organization diagnostic criteria for diabetes from 1999 or with diagnosis of T2D in clinical records; 2) > 18 years old; 3) volunteered to participate in the study. The diagnostic criteria for T2D were fasting plasma glucose levels ≥ 7.0 mmol/L and 2-hour plasma glucose levels ≥ 11.1 mmol/L [13]. These parameters have been widely used in clinical trials and nutrition surveys in China [14, 15]. Exclusion criteria were: (1) patients without or with limited civil capacity; (2) those with untreated diagnosed mental disorders; (3) those with difficulties in reading or understanding; (4) those with tumors or major organ dysfunction affecting their ability to maintain daily life or self-care; (5) pregnant women. The study received ethical approval from the Medical Ethics Committee of Qidong City People’s Hospital (approval number:2023-001-01), and informed consent was obtained from all participants.

### Questionnaire design

The questionnaire design was established based on previous studies [16–18] and *Obesity and Weight Management for the Prevention and Treatment of Type 2 Diabetes: Standards of Medical Care in Diabetes-2022*. A pilot study among 25 patients yielded a Cronbach’s α of 0.7736, indicating acceptable internal consistency.

The final Chinese questionnaire encapsulated four dimensions, i.e., demographic characteristics, knowledge, attitude, and practice. The demographic characteristics included the following 13 items: age, gender, residence, education, medical-related occupation, physical labor occupation, monthly income, medical insurance, course of T2D, medication of blood glucose control, hyperlipidemia, fatty liver, and screening for excess visceral fat.

The knowledge dimension comprised 14 questions. Scores of 1 point were allocated for correct responses to questions 1–12 and 14, whereas incorrect or unclear answers were scored 0 points. Additionally, the 13th question assessed the validity of the questionnaire. The total score ranged from 0 to 13. The attitude dimension comprised 10 questions, evaluated on a five-point Likert scale. Questions 2 to 4 were positively phrased and evaluated with “Strongly agree” (5 points) ranging to “Strongly disagree” (1 point), while the remaining inquiries were negatively phrased. The total score ranged from 10 to 50. The practice dimension featured 9 questions that were also evaluated on a five-point Likert scale. Questions 1, 2, 5, 6, 7, and 9 were formulated with positive orientation, while the remainder had negative orientation. The total score ranged from 9 to 45.

Participants were categorized into three tiers based on their respective scores in each dimension of knowledge, attitude, and practice: strong knowledge, positive attitude, and proactive practice (80–100%); moderate knowledge, neutral attitude, and moderate practice (60–79%); and inadequate knowledge, negative attitude, and inappropriate practice (below 60%) [16].

### Questionnaire distribution

The questionnaire was administered in both paper-based and web-based modalities. The former was disseminated through hospital or department heads, while the latter utilized the *“Sojump” platform (*https://www.wjx.cn/*)* and reached participants via doctor-patient WeChat groups (Tencent) and QR codes positioned at clinic entrances. A team of four specialized medical professionals assisted participants during the completion of the questionnaire, particularly addressing inquiries related to the questionnaire. Following data collection, the research team meticulously examined the quality of the gathered data and removed responses with conspicuous logical inconsistencies or repetitive patterns.

### Statistical analysis

The required sample for this study was calculated based on Cochran’s sample size estimation equation (n = z^2^pq/e^2^). In this formula, n is the number of participants required, z = 1.96 in 95% confidence interval, p = expected proportion, q = 1-p, and e is 5% of the margin of error. We have taken 50% as the expected proportion to obtain the maximum sample size, and the estimated sample size for the present study was 384.

SPSS 26.0 and AMOS (IBM, Armonk, NY, USA) were used for statistical analysis. Continuous variables were expressed as means ± standard deviations (SDs) and compared by analysis of variance (ANOVA) (including education, monthly income, medical insurance, and medication of blood glucose control) or independent sample T-test (including age, gender, residence, medical related occupation, physical labor occupation, course of diagnosis with type 2 diabetes mellitus, hyperlipidemia, fatty liver and screening for excess visceral fat). Moreover, the categorical variables were expressed as n (%). Pearson’s correlation analysis was conducted to assess the interrelationship between KAP dimensions. The correlation strength was divided into weak (abstract value of correlation < 0.40), moderate (0.40 ≤ abstract value of correlation < 0.60), and strong (abstract value of correlation ≥ 0.60). Structural equation modeling (SEM) was utilized to test the following hypotheses: (1) age, education, monthly income, fatty liver, and screening for excess visceral fat would influence knowledge; (2) knowledge would positively impact attitude; (3) knowledge would positively affect practice; and (4) attitude would positively influence practice. The root mean squared error of approximation (RMSEA), Tucker-Lewis index (TLI), standardized root mean squared residual (SRMR), and comparative fit index (CFI) were used to evaluate the fitting of the SEM. Besides, univariate and multivariate analyses were conducted to explore the relationships between KAP and their influential factors. The KAP was dichotomized using a cutoff of 60% of the total score in each dimension. The variables with *P* < 0.05 in univariate analysis were included in multivariate analysis. A two-sided *P* < 0.05 represented a significant difference.

## Results

### Demographic characteristics

Among 543 collected questionnaires, 40 (7.37%) were excluded due to invalid responses, resulting in 503 (92.63%) that met the required sample size. The mean age of the participants was 56.70 ± 11.44 years, and 54.67% were aged < 60 years. The majority were male (55.07%), residing in urban areas (57.46%), with an education level of junior high school/senior high school/technical secondary school (62.62%). Moreover, 95.63% of participants did not have medical-related occupations, and 93.64% had occupations that did not involve physical labor. The majority (84.49%) have been diagnosed with T2D for over a year, and 47.91% used oral hypoglycemic drugs for glucose control. Additionally, 42.15% also had hyperlipidemia, and 48.11% were diagnosed with fatty liver (Table 1).

**Table 1:** Demographic data of participants and KAP scores

Variables	N (%)/mean ± SD	Knowledge		Attitude		Practice	
		mean ± SD	P	mean ± SD	P	mean ± SD	P
**Total**	503	8.03 ± 3.525		31.88 ± 3.524		25.08 ± 2.830	
Age, years*	56.70 ± 11.44		< 0.001		< 0.001		0.088
< 60	275(54.67)	8.67 ± 3.40		33.58 ± 3.47		25.21 ± 2.89	
≥ 60	228(45.33)	7.27 ± 3.53		31.04 ± 3.41		24.93 ± 2.75	
Gender*			0.723		0.203		0.005
Male	277(55.07)	8.13 ± 3.33		32.06 ± 3.42		25.08 ± 2.96	
Female	226(44.93)	7.92 ± 3.76		31.65 ± 3.64		25.09 ± 2.68	
Residence*			< 0.001		< 0.001		0.017
Rural	214(42.54)	7.21 ± 3.50		30.80 ± 3.69		24.67 ± 3.10	
Urban	289(57.46)	8.64 ± 3.43		32.68 ± 3.18		25.38 ± 2.58	
**Education** ^**#**^			< 0.001		< 0.001		0.001
Primary school or below	91(18.09)	5.99 ± 3.77		30.58 ± 3.56		24.27 ± 2.67	
Junior high school/ Senior high school/ Technical secondary school	315(62.62)	7.90 ± 3.26		31.85 ± 3.42		25.14 ± 2.80	
Junior college or above	97(19.28)	10.40 ± 2.69		33.20 ± 3.40		25.64 ± 2.94	
Medical related occupation*			0.001		0.195		0.431
Yes	22(4.37)	10.45 ± 2.67		32.82 ± 4.29		25.55 ± 3.57	
No	481(95.63)	7.92 ± 3.52		31.84 ± 3.48		25.06 ± 2.79	
Physical labor occupation*			0.857		0.001		0.340
Yes	32(6.36)	7.94 ± 3.45		29.50 ± 4.57		25.66 ± 3.61	
No	471(93.64)	8.04 ± 3.53		32.04 ± 3.39		25.04 ± 2.77	
**Monthly income, yuan** ^**#**^			< 0.001		0.001		0.001
< 5000	328(65.21)	7.06 ± 3.42		31.52 ± 3.25		24.86 ± 2.61	
5000–10,000	129(25.65)	9.72 ± 3.12		32.87 ± 3.76		25.25 ± 2.99	
10,000–20,000	33(6.56)	10.18 ± 2.72		32.15 ± 4.11		25.88 ± 3.64	
> 20,000	13(2.58)	10.31 ± 1.97		30.38 ± 4.46		27.08 ± 3.35	
**Medical insurance** ^**#**^			0.162		0.997		0.101
Statutory health insurance	488(97.02)	7.99 ± 3.51		31.88 ± 3.49		25.05 ± 2.80	
Statutory health insurance combined with commercial insurance	11(2.19)	8.91 ± 4.21		31.91 ± 5.34		26.73 ± 3.41	
Uninsured	4(0.80)	10.75 ± 1.71		32.00 ± 2.16		25 ± 4.08	
Course of diagnosis with type 2 diabetes mellitus*			0.557		0.803		0.016
< 1 year	78(15.51)	7.72 ± 4.03		32.03 ± 3.51		25.60 ± 2.73	
> 1 year	425(84.49)	8.09 ± 3.43		31.85 ± 3.53		24.99 ± 2.84	
**Medication of blood glucose control** ^**#**^			0.630		0.098		0.122
Oral hypoglycemic drugs.	241(47.91)	7.88 ± 3.65		32.01 ± 3.39		25.27 ± 2.89	
Injecting insulin.	71(14.12)	8.21 ± 3.70		30.86 ± 4.14		25.38 ± 2.91	
Combined control	169(33.60)	8.28 ± 3.19		32.14 ± 3.45		24.69 ± 2.71	
None of the above	22(4.37)	7.32 ± 4.03		31.68 ± 3.05		25.09 ± 2.58	
Hyperlipidemia*			0.708		0.491		0.941
Yes	212(42.15)	8.15 ± 3.32		31.92 ± 3.39		25.05 ± 2.80	
No	291(57.85)	7.95 ± 3.67		31.85 ± 3.63		25.10 ± 2.86	
Fatty liver*			< 0.001		0.057		0.324
Yes	242(48.11)	9.09 ± 2.99		32.06 ± 3.87		25.18 ± 2.98	
No	261(51.89)	7.05 ± 3.70		31.71 ± 3.17		24.99 ± 2.69	
Screening for excess visceral fat*			< 0.001		0.222		0.872
Yes	138(27.44)	9.49 ± 2.87		32.12 ± 3.44		25 ± 2.96	
No	365(72.56)	7.48 ± 3.60		31.79 ± 3.56		25.11 ± 2.78	

*: Compared by independent sample T test; ^#^: Compared by one-way analysis of variance

### Knowledge

The average knowledge score of participants was 8.03 ± 3.525 (possible range: 0–13). Participants aged < 60 years (*P* < 0.001), residing in urban areas (*P* < 0.001), with an education of junior college or above (*P* < 0.001), having a medical-related occupation (*P* = 0.001), with a monthly income of > 20,000 yuan (*P* < 0.001), having a fatty liver (*P* < 0.001), and who underwent screening for excessive visceral fat (*P* < 0.003) were likely to achieve higher knowledge scores **(**Table 1**)**. Correct response rates in the knowledge section ranged from 28.63 to 89.86%. Notably, 89.86% correctly identified that caloric restriction and regular exercise enhance energy expenditure, leading to sustained and stable weight loss outcomes (K7). Conversely, only 28.63% of participants were aware that for patients with challenging weight management via lifestyle changes and medication, gastrectomy and gastric bypass surgery offer viable options (K12). Moreover, 35.79% of participants correctly understood that BMI within 18.5 ∽ 24 kg/m^2^ indicates a healthy weight range, while values exceeding 24 signify overweight status, and those exceeding 28 indicate obesity (K1) (Figure S1).

### Attitude

The participants exhibited an attitude score of 31.88 ± 3.524 (possible range: 10–50). Higher attitude scores were likely to be observed among participants aged < 60 years (*P* < 0.001), residing in urban areas (*P* < 0.001), possessing an education of junior college or above (*P* < 0.001), having occupations not requiring physical labor (*P* = 0.001), and earning monthly income within the range of 5,000–10,000 yuan (*P* = 0.001) (Table 1**)**. In the attitude section, the rates of positive responses varied from 9.74 to 63.62%. Notably, a significant proportion of participants (63.62%) had a favorable attitude towards recognizing that achieving weight control demands greater effort than healthy individuals **(A2)**. Conversely, a minority of participants (9.74%) concurred that no definite medication-induced side effects were associated with weight loss (A7). Moreover, only 13.92% concurred that dieting and exercising for weight control were not distressing (A6) (Figure S2).

### Practice

The mean practice score among participants was 25.08 ± 2.830 (possible range: 9–45). Participants with female gender (*P* = 0.005), urban residence (*P* = 0.017), education of junior college or above (*P* = 0.001), monthly income > 20,000 yuan (*P* = 0.001), and duration of T2D < 1 year (*P* = 0.016) were likely to have higher practice scores (Table 1). Variations in adherence to recommended practices were presented among participants, with proportions ranging from 4.37 to 80.52%. Most participants (80.52%) refrained from consuming additional meals to prevent hypoglycemia **(P3)**. In contrast, only a small fraction of participants (4.37%) would consider surgery if lifestyle changes and medications prove ineffective for weight control **(P9)**. Moreover, merely 5.56% of participants pursued weight control through methods such as intermittent fasting or ketogenic diets in their daily lives (P2) (Figure S3).

### Correlation analysis and SEM

Pearson’s correlation analysis revealed weak positive associations between knowledge and attitude (*r* = 0.295, *P* < 0.001), knowledge and practice (*r* = 0.131, *P* < 0.001), and attitude and practice (*r* = 0.140, *P* = 0.002) (Table 2).

**Fig. 1:**
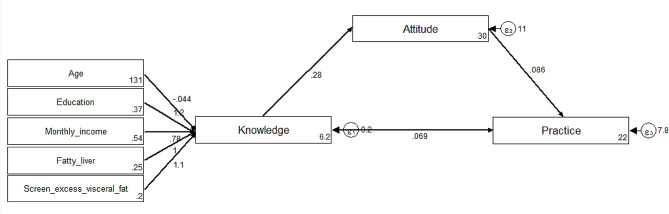
SEM showing the associations between sociodemographic characteristics and KAP. All variables are observed variables. Direction of causality is indicated by single-headed arrows

Moreover, the SEM exhibited good model fit (RMSEA = 0.076; SRMR = 0.042; TLI = 0.765; CFI = 0.870) (Table 3). The SEM outcomes revealed that knowledge had positive connections with attitude (β = 0.28, *P* < 0.001) and practice (β = 0.07, *P* < 0.001), and attitude was positively associated with practice (β = 0.09, *P* = 0.019). Age displayed a negative association with knowledge scores (β=-0.04, *P* = 0.001). Additionally, education (β = 1.20, *P* < 0.001), monthly income (β = 0.78, *P* < 0.001), diagnosis of fatty liver (β = 1.03, *P* = 0.002), and screening for excess visceral fat (β = 1.11, *P* = 0.002) (Table 4; Fig. 1).

**Table 2:** Pearson correlation analysis of knowledge, attitude and practice dimensions among participants

	Knowledge	Attitude	Practice
**Knowledge**	1		
**Attitude**	0.295 (*P* < 0.001)	1	
**Practice**	0.131 (*P* < 0.001)	0.140 (*P* = 0.002)	1

**Table 3:** Fitting of SEM

Indicators	Reference Standard	Actual results	Indication
RMSEA	< 0.08; good	0.076	good
SRMR	< 0.08 Good	0.042	good
TLI	> 0.8; good	0.765	good
CFI	> 0.8; good	0.870	good

RMSEA: root mean squared error of approximation, TLI: Tucker-Lewis index, CFI: comparative fit index, SRMR: standardized root mean squared residual

**Table 4:** The estimates of SEM

Model paths	Estimate	P
Knowledge → Attitude	0.28	< 0.001
Knowledge → Practice	0.07	0.063
Attitude → Practice	0.09	0.019
Age → Knowledge	-0.04	0.001
Education → Knowledge	1.20	< 0.001
Monthly income → Knowledge	0.78	< 0.001
Fatty liver → Knowledge	1.03	0.002
Screen for excess visceral fat → Knowledge	1.11	0.002

The results of univariate and multivariate analysis of KAP scores are shown in Table 5 and Table S1. Multivariate analysis showed that junior college education or above (OR = 7.25, 95%CI: 2.91, 18.0; *P* < 0.001), monthly income within 5000–10,000 yuan (OR = 1.98, 95%CI: 1.15, 3.40; *P* = 0.013), not having a fatty liver (OR = 2.26, 95%CI: 1.42, 3.60; *P* = 0.001), and not having screening for excess visceral fat (OR = 3.78, 95%CI: 2.37, 6.00; *P* < 0.001) were positively associated with knowledge score. Knowledge was positively associated with attitude (OR = 1.10, 95%CI: 1.03, 1.18; *P* = 0.003) and practice score (OR = 1.07, 95%CI: 1.00, 1.15; *P* = 0.040). Besides, age ≥ 60 years old (OR = 0.59, 95%CI: 0.37, 0.96; *P* = 0.035) and junior high school/ senior high school/ technical secondary school education (OR = 0.52, 95%CI: 0.27, 0.98; *P* = 0.045) were negatively associated with attitude. Urban residence (OR = 2.25, 95%CI: 1.39, 3.66; *P* = 0.001), and not having physical labor occupation (OR = 2.88, 95%CI: 1.30, 6.35; *P* = 0.009) were positively associated with attitude. Moreover, monthly income within 10,000–20,000 yuan (OR = 2.65, 95%CI: 1.14, 6.17; *P* = 0.023), > 20,000 yuan (OR = 4.20, 95%CI: 1.13, 15.60; *P* = 0.032), and having statutory health insurance combined with commercial insurance (OR = 5.04, 95%CI: 1.19, 21.30; *P* = 0.028) were positively associated with practice. However, not having physical labor occupation was negatively associated with practice (OR = 0.38, 95%CI: 0.17, 0.84; *P* = 0.017).

**Table 5:** Multivariate analysis for KAP

	Knowledge		Attitude		Practice	
	OR (95%CI)	P	OR (95%CI)	P	OR (95%CI)	P
**Knowledge score**	-	-	1.10(1.03,1.18)	0.003	1.07(1.00,1.15)	0.04
**Attitude score**	-	-	-	-	-	-
**Age, years**						
< 60	REF	REF	REF	REF		
≥ 60	1.21(0.77,1.90)	0.394	0.59(0.37,0.96)	0.035		
**Gender**						
Male						
Female						
**Residence**						
Rural	REF	REF	REF	REF		
Urban	1.16(0.74,1.81)	0.507	2.25(1.39,3.66)	0.001		
**Education**						
Primary school or below	REF	REF	REF	REF	REF	REF
Junior high school/ Senior high school/ Technical secondary school	1.93(1.08,3.45)	0.025	0.52(0.27,0.98)	0.045	1.44(0.76,2.72)	0.259
Junior college or above	7.25(2.91,18.0)	**< 0.001**	0.65(0.25,1.66)	0.369	1.21(0.53,2.75)	0.645
**Medical related occupation**						
Yes	REF	REF				
No	0.51(0.14,1.74)	0.283				
**Physical labor occupation**						
Yes			REF	REF	REF	REF
No			2.88(1.30,6.35)	0.009	0.38(0.17,0.84)	0.017
**Monthly income, yuan**						
< 5000	REF	REF			REF	REF
5000–10,000	1.98(1.15,3.40)	0.013			1.61(0.96,2.72)	0.07
10,000–20,000	2.02(0.65,6.26)	0.219			2.65(1.14,6.17)	0.023
> 20,000	5.30(0.63,44.1)	0.123			4.20(1.13,15.6)	0.032
**Medical insurance**						
Statutory health insurance					REF	REF
Statutory health insurance combined with commercial insurance					5.04(1.19,21.3)	0.028
Uninsured					2.17(0.27,16.9)	0.46
**Course of diagnosis with type 2 diabetes mellitus**						
< 1 year					REF	REF
> 1 year					0.65(0.37,1.13)	0.132
**Medication of blood glucose control**						
Oral hypoglycemic drugs.			REF	REF		
Injecting insulin.			0.58(0.31,1.08)	0.09		
Combined control			1.20(0.72,2.00)	0.477		
None of the above			0.58(0.21,1.59)	0.298		
**Hyperlipidemia**						
Yes						
No						
**Fatty liver**						
Yes	REF	REF			REF	REF
No	2.26(1.42,3.60)	0.001			1.28(0.84,1.97)	0.245
**Screening for excess visceral fat**						
Yes	REF	REF				
No	2.21(1.26,3.88)	0.005				

## Discussion

The present study revealed that diabetic patients had moderate knowledge, neutral attitudes, and inappropriate practices toward weight management. Furthermore, there were significantly positive correlations among KAP. Our study also elucidated associations between KAP scores and demographic variables, including age, education, and monthly income. These outcomes provide valuable guidance for devising healthcare interventions and educational initiatives to improve the knowledge, attitudes, and practices on weight management within the diabetic population.

Consistent with our findings, previous investigations in Bangladesh indicated that many T2D patients lacked awareness about optimal body weight, energy needs, and weight measurement methods [19]. Similarly, a study that included diabetic patients in Ethiopia reported moderate knowledge and neutral attitudes, coupled with limited adoption of lifestyle modifications such as adopting a healthy diet, achieving moderate weight loss, and engaging in regular physical activity [20]. These results emphasize the importance of bolstering education on weight management for diabetic patients in order to rectify gaps in knowledge, attitude, and practice.

In the knowledge dimension, the majority (89.86%) knew that combining caloric restriction with regular exercise was pivotal for increasing energy expenditure, resulting in consistent and steady weight loss. This heightened awareness could be attributed to widespread information regarding the importance of a balanced diet and physical activity in weight control from healthcare practitioners, media outlets, and educational campaigns [21, 22]. Conversely, only 28.63% of diabetic patients were aware of surgical alternatives available for individuals facing challenges in weight management through conventional approaches and medication. This observation suggested a gap in knowledge regarding the potential efficacy of surgical interventions such as gastrectomy and gastric bypass surgery. This disparity in awareness highlighted the need for improved education and dissemination of information on the range of available treatment options for weight management beyond lifestyle changes and medication. Additionally, the finding that only 35.79% of diabetic patients accurately comprehended BMI classifications highlights the necessity for enhanced patient education concerning BMI and its implications. This lack of awareness could hinder the patients’ perception of their weight status and understanding of associated health risks. Therefore, effective interventions should prioritize enhancing diabetic patients’ knowledge of key weight management concepts, encompassing the potential advantages of various treatments, the role of surgery in intricate cases, and BMI interpretation.

In the attitude dimension, the majority (63.62%) had a favorable perspective and acknowledged the heightened personal efforts required to achieve weight control compared to healthy individuals. This prevailing positive attitude potentially arose from enhanced awareness of the intricacies of weight management, which aligned with medical guidance that underscored the multifaceted nature of maintaining a healthy weight [23]. Conversely, the minority (9.74%) concurred that no definite medication-induced side effects were associated with weight loss. This incongruity in attitudes might stem from varying exposure levels to information about medication-related side effects. This viewpoint indicated potential misconceptions or lack of awareness of potential risks and consequences associated with specific weight loss medications. Through accurate and comprehensive education, participants could develop a well-informed understanding of potential risks and benefits associated with weight loss interventions, particularly those involving medications. Furthermore, 13.92% of participants agreed that their diet and exercise experiences for weight control have been free from distress. This relatively modest concurrence rate implied that the majority likely encountered challenges in adhering to weight management strategies. Comprehensive support strategies are needed to address this issue, facilitate effective weight control, and alleviate distress or discomfort associated with lifestyle modifications [24].

In practice, the majority (80.52%) exhibited a strong willingness to manage weight by refraining from extra meals to prevent hypoglycemia. This adherence aligned with the importance of stable blood glucose levels in diabetes management, which is encouraging from a health standpoint [25]. Nevertheless, the minority (4.37%) expressed willingness to consider surgical interventions if conventional methods like lifestyle changes and medications proved ineffective for weight control, which suggested hesitancy towards surgical options, potentially due to concerns about invasiveness or perceived risks. This highlighted the need for comprehensive education and discussions about the benefits and risks of surgical interventions for weight management. Furthermore, only 5.56% reported adopting weight control methods like intermittent fasting or ketogenic diets in their daily routine. This modest engagement might reflect varied perceptions on the suitability and sustainability of these approaches since individual preferences, metabolic responses, and long-term feasibility influence the appropriateness of these methods [26–28]. These findings underscored the need for a patient-centered approach to diabetes and weight management. Healthcare professionals should emphasize established practices like avoiding extra meals for hypoglycemia prevention and discuss alternative strategies such as surgical interventions and specialized diets. Based on the above items, improving diabetic patients’ KAP regarding weight management may require a multifaceted approach that includes enhancing awareness of surgical alternatives, providing comprehensive education on BMI classifications and medication-induced side effects, addressing emotional distress, promoting open discussions about surgical options, and offering a range of evidence-based weight control methods. Such efforts can empower patients to make informed decisions and participate in weight management.

The positive correlation of knowledge with attitude and practice emphasized the need for targeted educational programs to enhance positive attitudes and promote weight management practices among diabetic patients. Also, the positive correlation between attitude and practice aligned with the theory of planned behavior, stating that attitudes toward the behavior can impact engagement [29]. However, the correlation analysis indicated the correlations were weak, indicating other potential factors might impact their KAP. Several influential factors of KAP were further identified by univariate and multivariate logistic regression analysis. Advanced age might contribute to reduced awareness and understanding of weight management, possibly due to generational differences in information access and health concept receptivity [30]. Patients with junior college or higher education had a positive association with knowledge, highlighting the key role of education in providing requisite information and cognitive abilities for comprehending intricate health concepts, including effective weight management. Moreover, increased financial resources could enhance access to health-related information and foster a more comprehensive grasp of weight management principles.

The present study has several limitations. First, although the sample size met the requirement, the samples were collected from a single province, potentially constraining generalizability. Nevertheless, these findings offered valuable perspectives on the current KAP of diabetic patients toward weight management. Second, social desirability bias could affect the KAP outcomes, potentially resulting in inflated scores [31]. Diabetic patients might have provided socially favorable responses, potentially diverging from their genuine behaviors and knowledge. Thirdly, the response rate could not be calculated due to the open questionnaire collection methods.

In conclusion, diabetic patients exhibited moderate knowledge, neutral attitudes, and inappropriate practices regarding weight management. To enhance their KAP, targeted educational interventions were recommended, particularly for older people, individuals with limited education, and those with lower monthly incomes. Interventions targeting weight management may serve as effective entry points for broader health promotion strategies. Initiatives focusing on weight management could be leveraged to impart comprehensive health education, addressing weight-related concerns and fostering a holistic understanding of health. Besides, public health programs aiming to enhance KAP in communities could consider incorporating weight management components. By integrating weight management strategies into existing health promotion initiatives, these programs may tap into individuals’ existing motivations and interests, potentially yielding more comprehensive and sustained health improvements.

### Electronic supplementary material

Below is the link to the electronic supplementary material.

Supplementary Material 1

Supplementary Material 2

## Data Availability

All data generated or analysed during this study are included in this published article.
