# Supplementary material for: Knowledge, attitude, and practice toward weight management among diabetic patients in Qidong City, Jiangsu Province
Source: BMC Public Health. 2024 Mar 29;24:922. doi: 10.1186/s12889-024-18392-2 (PMC10979591; doi:10.1186/s12889-024-18392-2)
Supplement: Supplementary file 1 — Supplementary Material 1 [file 12889_2024_18392_MOESM1_ESM.docx]

Supplementary Figure 1. Distribution of knowledge dimension responses.

Supplementary Figure 2. Distribution of attitude dimension responses.

Supplementary Figure 3. Distribution of practice dimension responses.

Supplementary Table 1. Univariate analysis for KAP.

|  | **Knowledge** | | **Attitude** | | **Practice** | |
| --- | --- | --- | --- | --- | --- | --- |
|  | **OR (95%CI)** | **P** | **OR (95%CI)** | **P** | **OR (95%CI)** | **P** |
| **Knowledge** |  |  | 1.12(1.05,1.19) | **<0.001** | 1.12(1.06,1.19) | **<0.001** |
| **Attitude** |  |  |  |  | 1.03(0.98,1.09) | 0.175 |
| **Age, years** |  |  |  |  |  |  |
| <60 | REF | REF | REF | REF | REF | REF |
| ≥60 | 0.66(0.46,0.95) | **0.027** | 0.52(0.34,0.79) | **0.002** | 0.70(0.47,1.04) | 0.085 |
| **Gender** |  |  |  |  |  |  |
| Male | REF | REF | REF | REF | REF | REF |
| Female | 0.93(0.65,1.33) | 0.716 | 0.76(0.50,1.15) | 0.201 | 1.07(0.72,1.58) | 0.714 |
| **Residence** |  |  |  |  |  |  |
| Rural | REF | REF | REF | REF | REF | REF |
| Urban | 2.09(1.45,3.00) | **<0.001** | 2.66(1.74,4.06) | **<0.001** | 1.02(0.69,1.51) | 0.891 |
| **Education** |  |  |  |  |  |  |
| Primary school or below | REF | REF | REF | REF | REF | REF |
| Junior high school/ Senior high school/ Technical secondary school | 2.42(1.49,3.93) | **<0.001** | 1.23(0.73,2.07) | 0.416 | 1.76(0.98,3.16) | 0.054 |
| Junior college or above | 13.0(6.21,27.4) | **<0.001** | 2.72(1.30,5.69) | **0.008** | 2.68(1.37,5.23) | **0.004** |
| **Medical related occupation** |  |  |  |  |  |  |
| Yes | REF | REF | REF | REF | REF | REF |
| No | 0.30(0.10,0.91) | **0.034** | 0.93(0.33,2.59) | 0.899 | 0.46(0.19,1.10) | 0.085 |
| **Physical labor occupation** |  |  |  |  |  |  |
| Yes | REF | REF | REF | REF | REF | REF |
| No | 0.97(0.47,2.02) | 0.95 | 3.07(1.48,6.36) | **0.002** | 0.43(0.20,0.89) | **0.023** |
| **Monthly income, yuan** |  |  |  |  |  |  |
| <5000 | REF | REF | REF | REF | REF | REF |
| 5000-10000 | 3.59(2.26,5.70) | **<0.001** | 1.12(0.68,1.83) | 0.636 | 2.00(1.28,3.11) | **0.002** |
| 10000-20000 | 6.09(2.29,16.1) | **<0.001** | 0.97(0.42,2.24) | 0.953 | 3.28(1.58,6.82) | **0.001** |
| >20000 | 13.0(1.68,101.) | **0.014** | 0.36(0.11,1.11) | 0.077 | 7.85(2.35,26.2) | **0.001** |
| **Medical insurance** |  |  |  |  |  |  |
| Statutory health insurance | REF | REF | REF | REF | REF | REF |
| Statutory health insurance combined with commercial insurance | 1.91(0.50,7.30) | 0.341 | 0.54(0.15,1.89) | 0.341 | 6.97(1.82,26.6) | **0.005** |
| Uninsured |  |  |  |  | 2.61(0.36,18.7) | 0.339 |
| **Course of diagnosis with type 2 diabetes mellitus** |  |  |  |  |  |  |
| <1 year | REF | REF | REF | REF | REF | REF |
| >1 year | 1.27(0.78,2.06) | 0.33 | 1.03(0.58,1.81) | 0.91 | 0.59(0.35,0.98) | **0.042** |
| **Medication of blood glucose control** |  |  |  |  |  |  |
| Oral hypoglycemic drugs. | REF | REF | REF | REF | REF | REF |
| Injecting insulin. | 1.07(0.62,1.83) | 0.805 | 0.53(0.29,0.94) | **0.031** | 1.68(0.97,2.92) | 0.062 |
| Combined control | 1.06(0.71,1.58) | 0.771 | 1.14(0.70,1.85) | 0.579 | 0.75(0.48,1.18) | 0.227 |
| None of the above | 0.48(0.19,1.17) | 0.108 | 0.61(0.24,1.59) | 0.32 | 0.91(0.34,2.43) | 0.861 |
| **Hyperlipidemia** |  |  |  |  |  |  |
| Yes | REF | REF | REF | REF | REF | REF |
| No | 1.19(0.83,1.71) | 0.336 | 1.23(0.80,1.87) | 0.333 | 0.84(0.57,1.25) | 0.412 |
| **Fatty liver** |  |  |  |  |  |  |
| Yes |  |  |  |  |  |  |
| No | 3.49(2.39,5.09) | **<0.001** | 0.98(0.65,1.48) | 0.956 | 1.74(1.17,2.57) | **0.005** |
| **Screening for excess visceral fat** |  |  |  |  |  |  |
| Yes | REF | REF | REF | REF | REF | REF |
| No | 3.78(2.37,6.00) | **<0.001** | 1.32(0.82,2.13) | 0.249 | 1.11(0.72,1.70) | 0.625 |
